# Gold nanoparticle decorated post-synthesis modified UiO-66-NH_2_ for A^3^-coupling preparation of propargyl amines

**DOI:** 10.1038/s41598-023-35848-4

**Published:** 2023-06-03

**Authors:** Leila Mohammadi, Reza Taghavi, Mojtaba Hosseinifard, Mohammad Reza Vaezi, Sadegh Rostamnia

**Affiliations:** 1 Department of Nano Technology and Advanced Materials, Materials and Energy Research Center, Karaj, Iran; 2 Organic and Nano Group (ONG), Department of Chemistry, Iran University of Science and Technology (IUST), PO BOX 16846-13114, Tehran, Iran; 3 Department of Energy, Materials and Energy Research Center, Karaj, Iran

**Keywords:** Chemistry, Catalysis, Organic chemistry

## Abstract

In this report, the novel UiO‑66‑NH_2_ based-MOF(Zr) catalytic system which further modified with nitrogen-rich organic ligand (5-aminotetrazole) using post synthetic modification (PSM) approach has been prepared here as an efficient catalyst to promote the A^3^-coupling preparation of propargyl amines in green aquatic media. This newly highly efficient catalyst was synthesized upon Zr-based MOF (UiO‑66‑NH_2_) which successfully functionalized with 2,4,6‑trichloro‑1,3,5‑triazine (TCT) and 5‑aminotetrazole, following through stabilization of gold metal (Au) nanopartilces. The addition of *N*-rich organic ligand through post-synthesis modification which can be assisted to stabilize the bister and stable gold nanoparticles caused to unique structure of the final composite in favor of the progress of the A^3^ coupling reaction. Also several strategies comprising XRD, FT-IR, SEM, BET, TEM, TGA, ICP, EDS and elemental mapping analyzes, were used to indicate the successful preparation of the UiO-66-NH_2_@ Cyanuric Chloride@ 5-amino tetrazole/Au-NPs. The results of productivity catalyst are accomplished in good to excellent yields for all sort of reactions under mild conditions which is a proof of superior activity heterogeneous catalyst containing Au-nanoparticles. In addition, the suggested catalyst represented excellent reusability with no remarkable loss in activity up 9 sequential runs.

## Introduction

Synthetic progresses including three or more well-defined reactants to generate an ingredient which contains noteworthy fragments of all reactants, exquisitely all atoms as Multi-component reactions (MCRs) are concentrated^1–3^. Additionally, this class of responses suggests a higher level of atomic efficiency because of time-saving isolation, as well as filtration of synthetic intermediates. Lately, a multitude of MCRs, A^3^-type coupling reactions, involving amines, aldehydes by terminal alkynes, directly in a one-pot process, as a robust, valuable, and specific method for a set of complex molecules by noteworthy biological properties through the diverse elementary, as well as remarkable pioneers have been efficiently developed^4–7^. In the last few decades, the synthesis of propargylamines have been facinated attention, because of their great use as a pharmaceutical vital drug in medical field^8–10^. Preparation of propargylamines through the A^3^-coupling reaction gained broad attention, due to economical, easy availability, and adaptability, have been effectively developed in research as compatibility scaffolds of chemical methods and key intermediate drugs to combine biological-active nitrogenous scaffolds including, polyfunctional amino compounds, oxotremorine analogs, *β*-lactams, pyrrolidines, oxazoles, indolysines, pyrroles, triazolodiazepines as well as pesticides, insecticides, and also medicinal substances for treatment of Alzheimer's and Parkinson's disease^11–14^.

Activation of the C-H bond of the terminal alkyne is the main reason for the utilization of metal NPs for this reaction. Ag^15^, Cu^16^, and Au^17^ are some utilized metal NPs to promote this reaction. Gold nanoparticles, as one of the most widely utilized metals has been effectively applied in A^3^-coupling reactions due to its economical, availability and high potential for reactivity^18–20^. There are various reports regarding utilizing Au NPs as catalysts to promote the A^3^-coupling reaction^8,21^. Due to the ultrahigh sensitivity of the catalytic properties of the gold NPs to their size and shape, the utilization of supports for loading the NPs and preventing their aggregation is of great importance^22–24^. Porous organic polymers (POPs), coordination organic frameworks (COFs), silica mesoporous materials, and metal–organic frameworks are some of the utilized nano and micro-materials to prevent aggregation of Au NPs and modulate their catalytic properties^25–28^.

One of the most popular materials for the heterogenization of metal NPs is metal–organic frameworks (MOFs), a family of porous materials with tunable chemical and physical properties^29,30^. The designable structure of these materials leads to their broad application in various branches of chemistry, including catalysis, photocatalysis, electrocatalysis, adsorption, separation, drug delivery, and organic transformation. Moreover, post-synthesis modification (PSM) allows for the extensive tuning of the chemical and physical characteristics in MOF by implanting a wide variety of organic and inorganic functionalities^31,32^.

Synthesis of organic compounds in green aquatic solvents conditions is a specific topic in today’s scientific community. Accomplishments within the field of green chemistry have opened up an awesome prospect for more prominent impacts on the efficiency and performance of chemicals whereas reducing their adverse effects, also facilitating the safety and wellbeing of incorporating mild conditions, as well expanding the scope of various organic reactions^33,34^.

Metal–organic frameworks (MOFs), known as a class of reticular nanoporous materials with designable chemical and physical characteristics, are one of the most commonly used materials for the heterogenization of metal NPs. While the metal NPs act as active catalytic centers, MOFs can add numerous features and functionalities to the composite^35,36^. A large number of organic and inorganic functionalities can be implanted in MOF via the post-synthesis modification (PSM) process, resulting in a high degree of chemical and physical properties tunability. Such a tunable structure of MOFs leads to their wide utilization in a plethora of applications. Sensing, drug delivery, catalysis, photocatalysis, electrocatalysis, adsorption, separation, organic transformation, etc., are just a portion of their applications. Judicious implantation of organic additives to the MOFs structure through PSM can boost their performance toward a specific application. Moreover, the PSM of MOFs can elevate their water resistance, which is one of their main drawbacks^37–41^.

UiO-66-NH_2_, with a Zr_6_O_4_(OH)_4_ inorganic cluster, is best known for its inherent stability in aquatic mediums due to the presence of Zr^4+^ ions in its inorganic clusters^42^. The high affinity of the negative carboxylic acids to the highly positive Zr^4+^ ions, along with the crowded secondary building block (SBU), hindering the accessibility of H_2_O molecules to the zirconia cluster, led to the exceptional stability of UiO-66 structure^43,44^. High stability, inherent open metal sites (OMSs), high surface area, and the presence of the amino group make UiO-66-NH_2_ a perfect candidate for the PSM. Amino groups of the organic linker can act as a binding center to modify the UiO-66-NH_2_ electronic structure for the desired applications^45^.

Here, we synthesized a UiO-66-NH_2_ MOF and employed a PSM procedure to modify its structure with *N*-rich organic ligand (5-aminotetrrazole). The obtained UiO-66-NH_2_@cyanuric chloride@5-amino tetrazole was used as support to load and heterogenize the Au NPs. Finally, we employed the UiO-66-NH_2_@cyanuric chloride@5-amino tetrazole/Au_NPs_ as a catalyst for the promotion of the A^3^-coupling preparation of a series of propargyl amines. The designed catalyst exhibits a superior catalytic performance due to the modulation of the microenvironment of Au NPs via PSM of MOF. The suggested catalyst exhibits increased catalytic performance as a consequence of the PSM. Moreover, the proposed catalyst showed excellent recyclability up to 9 cycles. The addition of *N*-rich organic ligand through post-synthesis modification which can be assisted to stabilize the bister and stable gold nanoparticles caused to unique structure of the final composite in favor of the progress of the A^3^ coupling reaction.

## Experimental

All the applied materials and reagents in this work were purchased from merk and Sigma-Aldrich companies and used without further purification.

### Synthesis of UiO-66-NH_2_

To synthesize UiO-66-NH_2_, (2.05 g, 0.011 mol) 2-aminoterephthalic acid (NH_2_-BDC) was poured to a three-neck round-bottom flask containing 65 mL *N*, *N*-Dimethylformamide anhydrous (dry DMF) under constant stirred for 10 min at room temperature. Then, (2.65 g, 0.011 mol) zirconium tetrachloride (ZrCl_4_) and 55 mL DMF were added to this solution. This solution was charged with 2.5 mL of concentrated HCl, and the reaction was carried out under reflux condition at inert argon atmosphere at 130 °C for 24 h. Finally, the yellow powder was separated from the reaction blend and washed with DMF and methanol three times. MOF was activated by soaking the as-synthesized UiO-66-NH_2_ in methanol for two days and drying under a vacuum at 60 °C.

### Synthesis of UiO-66-NH_2_@cyanuric chloride

Post-synthesis modification of UiO-66-NH_2_ was performed through the following procedure. First, 0.6 g cyanuric chloride was dissolved in 40 mL dry DMSO at 25 °C. Next, 1 g UiO-66-NH_2_ was added to the obtained solution and stirred for 24 h at 60 °C. the resulting solid was filtered and washed with DMSO for purification.

### Synthesis of UiO-66-NH_2_@cyanuric chloride@5-amino tetrazole

To a flask containing dry acetonitrile (30 ml), 1 g UiO-66-NH_2_@cyanuric chloride was added and stirred for 10 min. To another beaker containing 30 mL dry acetonitrile, 1 g of 5-amino tetrazole was added, stirred until it was completely dissolved. After that, the contents of 5-amino tetrazole dissolved in dry acetonitrile was poured to UiO-66-NH_2_@cyanuric chloride and stirred for 24 h at 60 °C. Finally, the precipitate was filtered, washed with acetonitrile, and dried at 45 °C.

### Synthesis and stabilization of gold NPs

First, 0.2 g of as-synthesized UiO-66-NH_2_@cyanuric chloride@5-aminotetrazole was added to the flask containing 35 mL of distilled water. Then, 0.045 g of Chloroauric acid (HAuCl_4_) was dissolved in 5 mL distilled water and added to the above balloon dropwise under vigorous stirring for 5 h at ambient temperature. Following, the reaction mixture was charged with 0.3 ml of freshly prepared hydrazine hydrate solution (3 drops of hydrazine hydrate in 3 ml of deionized water) and kept stirring at r.t for another 24 h. Consequently, the reaction mixture was separated using a 9000 rpm centrifuge, washed once with distilled water, and dried in an oven.

### General procedure for the preparation of the propargyl amines

To investigate the catalytic performance of the proposed catalyst, the progress of the A^3^-coupling preparation of propargyl amines from the corresponding aldehydes (1 mmol), phenylacetylene (1.1 mmol), and the secondary amines (1 mmol) under the optimum condition was monitored (Table 2). Purification of the synthesized propargyl amines was performed by plate-chromatography. The chemical structure of the synthesized propargyl amines was probed by ^1^H NMR and ^13^C NMR (Supplementary information).

## Results and discussion

**Scheme 1.**
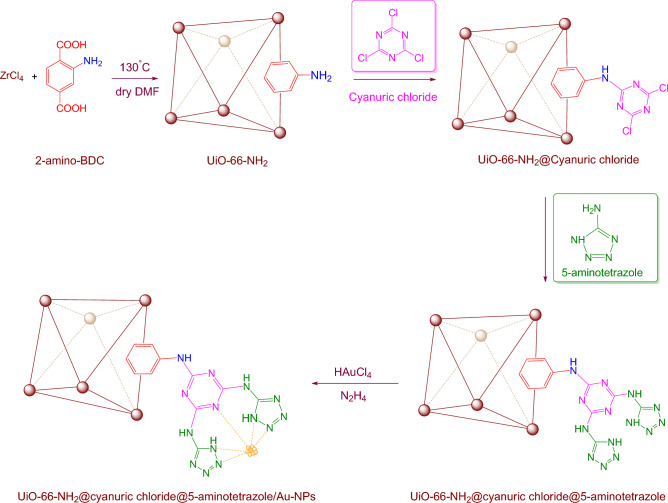
Schematic synthesis of gold nanoparticle immobilized on UiO-66-NH_2_@cyanuric chloride.

Following our previous attempts to develop facile and sustainable methodologies for developing various organic reactions, in this report, we introduce a highly efficient and recyclable gold-based catalyst for the promotion of the A^3^-coupling reaction for the preparation of the medicinally important propargyl amine derivatives from an aldehyde, an amine, and a terminal alkyne as the starting materials. First, the UiO-66-NH_2_ was prepared and modified with two *N*-rich organic ligands. A step-by-step PSM method was applied to introduce the cyanuric chloride and 5-amino tetrazole to the frameworks. Then, synthesized UiO-66-NH_2_@cyanuric chloride@5-amino tetrazole was used as support for the heterogenization of the Au NPs (Scheme 1). Finally, the manufactured UiO-66-NH_2_@cyanuric chloride@5-amino tetrazole/Au_NPs_ was employed as a catalyst for the A^3^-coupling preparation of a series of propargyl amines.

**Figure 1:**
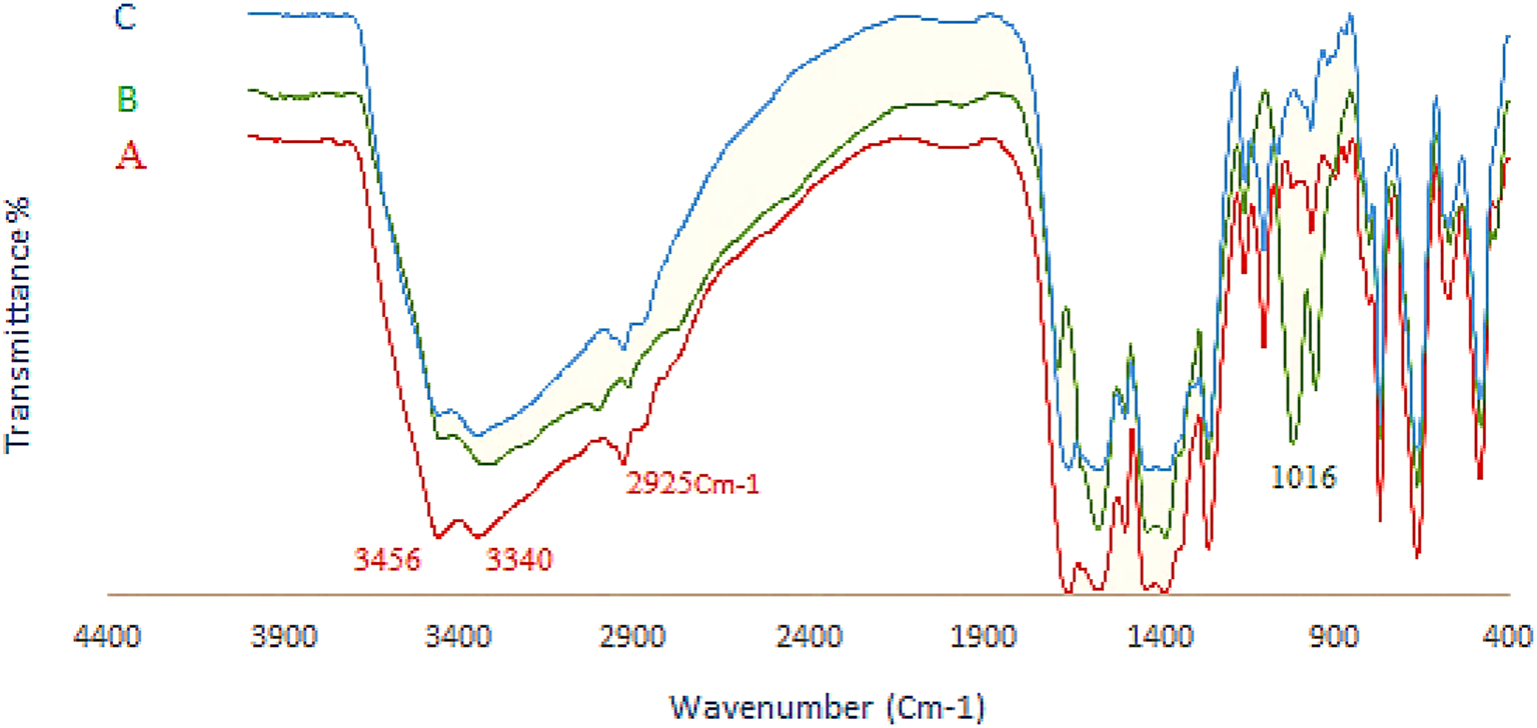
FT-IR spectra of (**A**) UiO-66-NH_2_, (**B**) UiO-66-NH_2_@cyanuric chloride, and (**C**) UiO-66-NH_2_@cyanuric chloride@5-amino tetrazole.

The FT-IR spectra of UiO-66-NH_2,_ UiO-66-NH_2_@cyanuric chloride, and UiO-66-NH_2_@cyanuric chloride@5-amino tetrazole are represented in figure. The F-IR spectra of bare UiO-66-NH_2_ (Fig. 1A) shows a broad peak at 1500–1600 and 3300–3500 cm^-1^, regarding the free and uncoordinated NH_2_ groups. Stretching vibrations of the C-N bonding of the H_2_BDC-NH_2_ are showing themselves at 1261 and 1338 cm^-1^. Cyanuric chloride modified MOF shows less intensity for the broad peak of the NH_2_ group at 3300–3500 cm^-1^, indicating less free and available uncoordinated NH_2_. Moreover, a new sharp peak at 1016 cm^-1^, which assigned the presence of cyanuric chloride in the compound (Fig. 1B). The same peak at 3300–3500 cm^-1^ disappeared from the IR spectra of the UiO-66-NH_2_@cyanuric chloride@5-amino tetrazole due to the coordination of 5-amino tetrazole to the cyanuric chloride. And also, the peak related to 1016 cyanuric chloride is no longer observed, which is a proof of the presence of 5-aminotetrazol (Fig. 1C). Other characteristic peaks of various parts of the composite overlapped, and other characterization methods were used to prove the formation of the catalyst.

**Figure 2:**
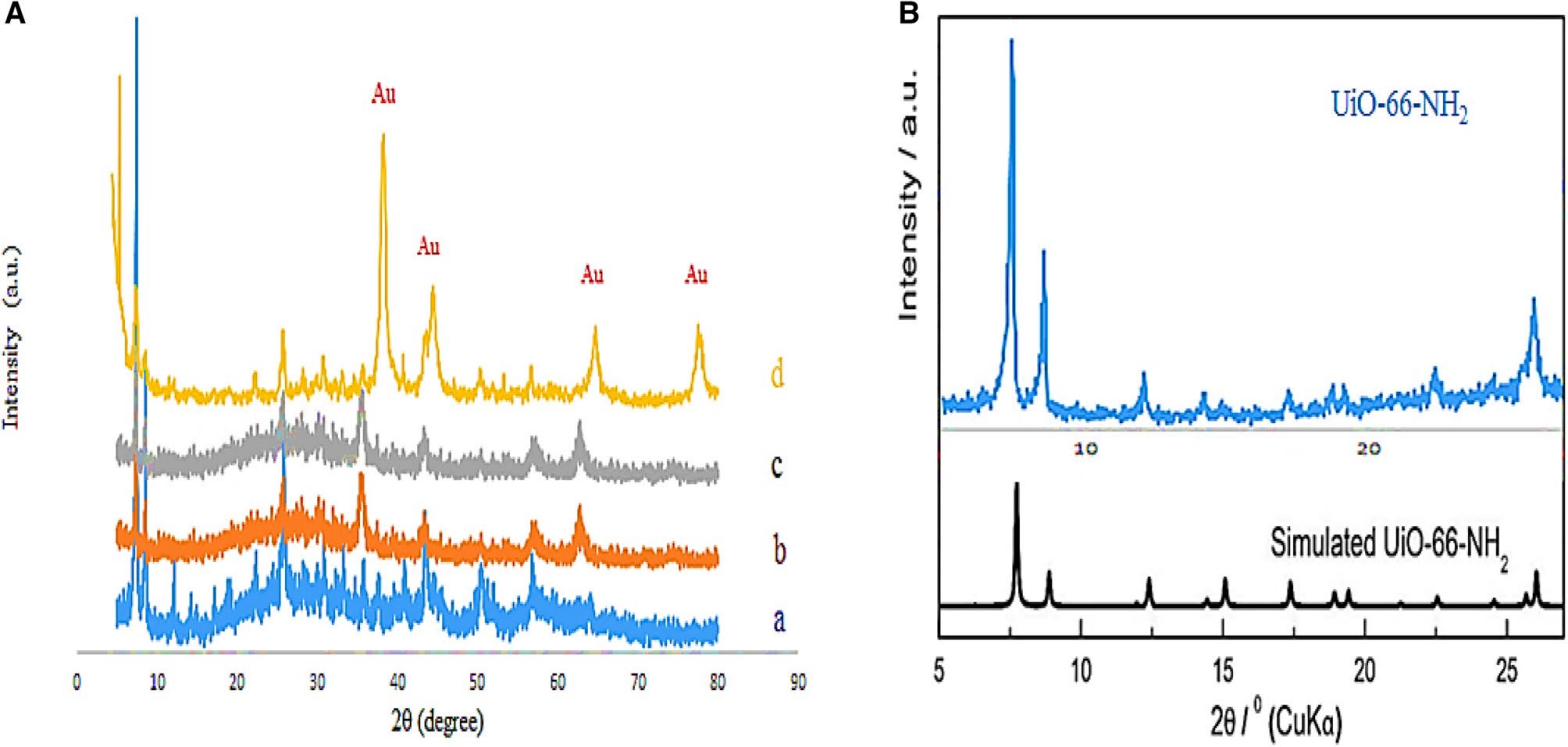
(**A**) XRD spectra of (a) UiO-66-NH_2_, (b) UiO-66-NH_2_@cyanuric chloride, (c) UiO-66-NH_2_@cyanuric chloride@5-amino tetrazole, and (d) UiO-66-NH_2_@cyanuric chloride@5-amino tetrazole/Au_NPs_. (**B**) The simulated XRD of UiO-66-NH_2_ to verify the successful preparation of UiO-66-NH_2_.

The crystalline structure of as-synthesized UiO-66-NH_2_, UiO-66-NH_2_@cyanuric chloride, UiO-66-NH_2_@cyanuric chloride@5-amino tetrazole, UiO-66-NH_2_@cyanuric chloride@5-amino tetrazole/Au_NPs_, and the simulated XRD of UiO-66-NH_2_ in order to verify the successful preparation of UiO-66-NH_2_ are investigated by XRD technique (Fig. 2). The XRD pattern of bare UiO-66-NH_2_ reveals all the supposed characteristics which prove its crystallinity and successful synthesis (Fig. 2Aa). The XRD pattern of cyanuric chloride modified UiO-66-NH_2_ exhibits no noticeable change in comparison to the bare MOF, which proves that post-synthesis modification process did not affect the crystallinity (Fig. 2Ab). The XRD pattern of UiO-66-NH_2_@cyanuric chloride@5-amino tetrazole exhibit minor broadening in the XRD peaks of MOF, which shows that further modification leads to a minor loss of crystallinity, proving the successful modification (Fig. 2Ac). After the composition of UiO-66-NH_2_@cyanuric chloride@5-amino tetrazole with gold NPs, four new peaks appear, which correspond to standard Bragg reflections (111), (200), (220), and (311) of face centers cubic lattice of gold NPs (Fig. 2Ad). This spectra also exhibits all the characteristics of UiO-66-NH_2_, with a minor shift to higher 2θ which is a natural result of the composition, proving that the MOF preserve its crystalline structure through all the synthesis process. Moreover, the XRD pattern of the simulated XRD of UiO-66-NH_2_ in order to verify the successful preparation of UiO-66-NH_2_ reveals all the supposed characteristics which prove its crystallinity and successful synthesis (Fig. 2B).

**Figure 3:**
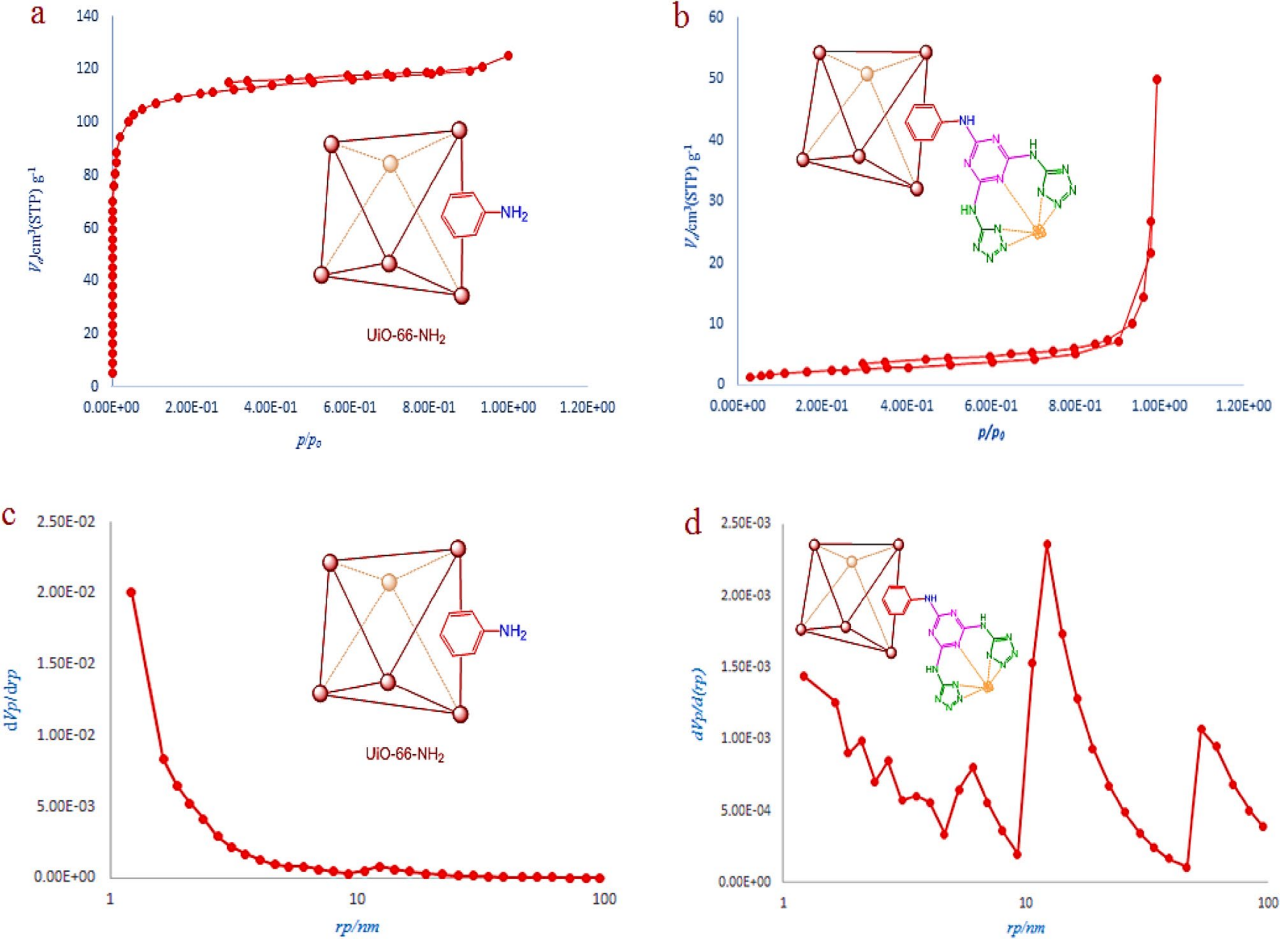
The BET (**a**) and BJH (**c**) of of UiO-66-NH_2_. The BET (**b**) and BJH (**d**) of UiO-66-NH_2_@cyanuric chloride@5-amino tetrazole.

N_2_ adsorption–desorption experiment was conducted for bare and modified UiO-66-NH_2_ at 77 K to determine the porosity of the MOF. Brunauer–Emmett–Teller (BET) was conducted to calculate the surface area of the samples (Fig. 3a). The adsorption–desorption isotherm of the bare UiO-66-NH_2_ shows a type I isotherm, which suggests the microporosity of its structure, with a surface area of 375 cm^3^ g^-1^. The BJH plot of UiO-66-NH_2_ confirms the microporosity of its matrix. This plot shows only one type of micropore in the bare MOF structure with a pore diameter of 1.21 nm (Fig. 3c). The modified MOF shows a dramatic decrease in surface area from 375 to 149 cm^3^ g^-1^. This result is a consequence of the successful PMS of MOF, in which the cyanuric chloride and 5-amino tetrazole filled the pores of UiO-66-NH_2_ and reduced its surface area (Fig. 3b, d).

**Figure 4:**
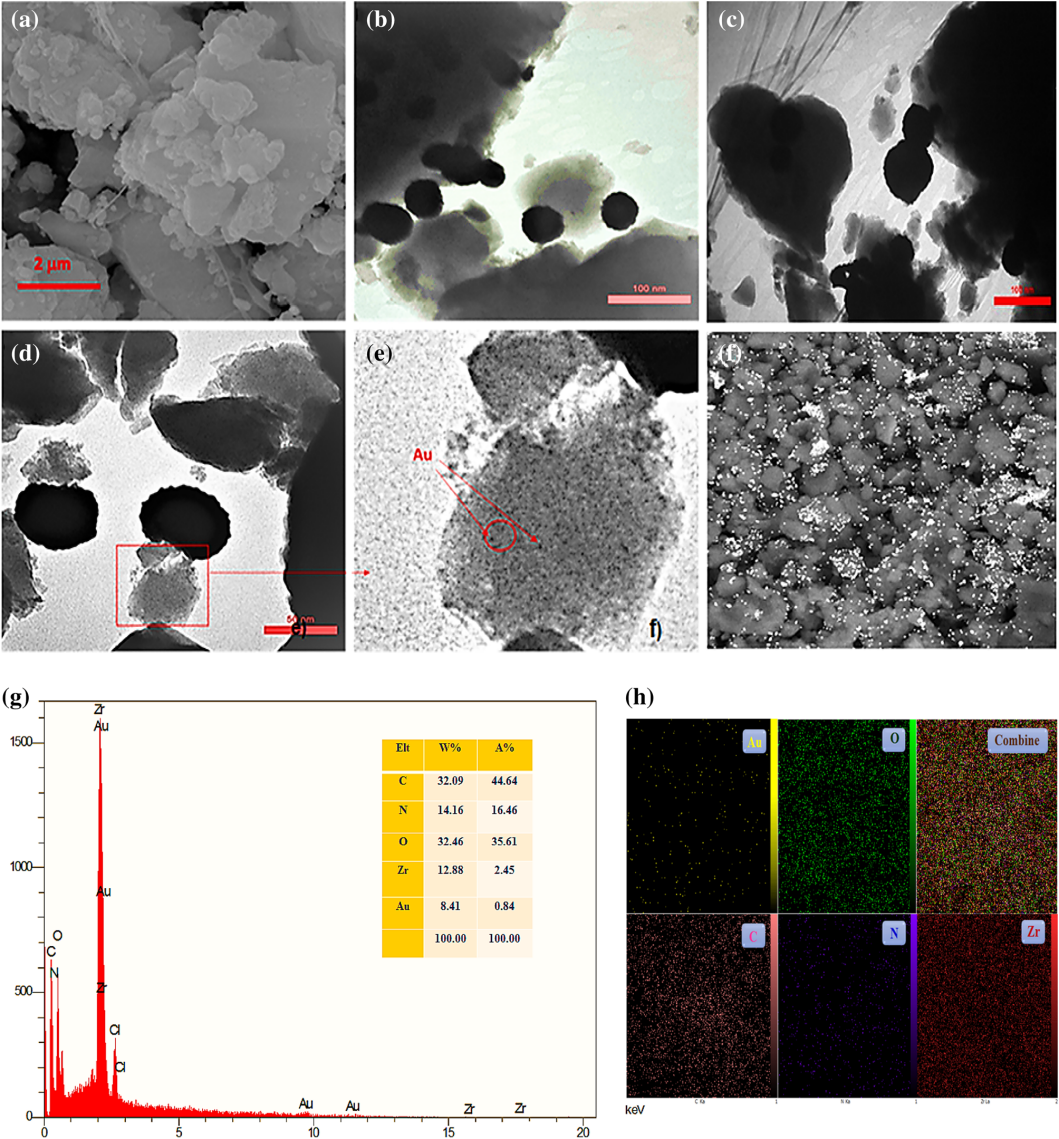
(**a**) SEM, (**b**, **c**) TEM, (**d**) magnified TEM for selected part of (**c**). (**e**) SEM-Mapping and (**f**) STEM of UiO-66-NH_2_@cyanuric chloride@5-amino tetrazole/Au_NPs_, (**g**) EDS, (**h**) Mapping.

The morphology and surface structure of UiO-66-NH_2_@cyanuric chloride@5-amino tetrazole/Au_NPs_ are investigated by SEM and TEM techniques (Fig. 4a–f). SEM images show the coarse surface of UiO-66-NH_2_ after the post-synthesis modification process, which is a natural result of the PSM and composition with the gold NPs (Fig. 4a). Because of the obtained results from the BET, showing that the pores of the MOF are laden due to the PSM, we try to use the surface of the modified MOF as a stand to load the Au NPs. In this context, we used hydrazine hydrate as a reductant, which results in the formation of bigger NPs compared to other reductants such as NaBH_4_, hoping to form Au_NPs_ over the surface of modified MOF. TEM image exhibits the presence of well-dispersed Au NPs over the surface of UiO-66-NH_2_ (Fig. 4b–d) EDS and elemental Mapping (Fig. 4g, h).

**Figure 5:**
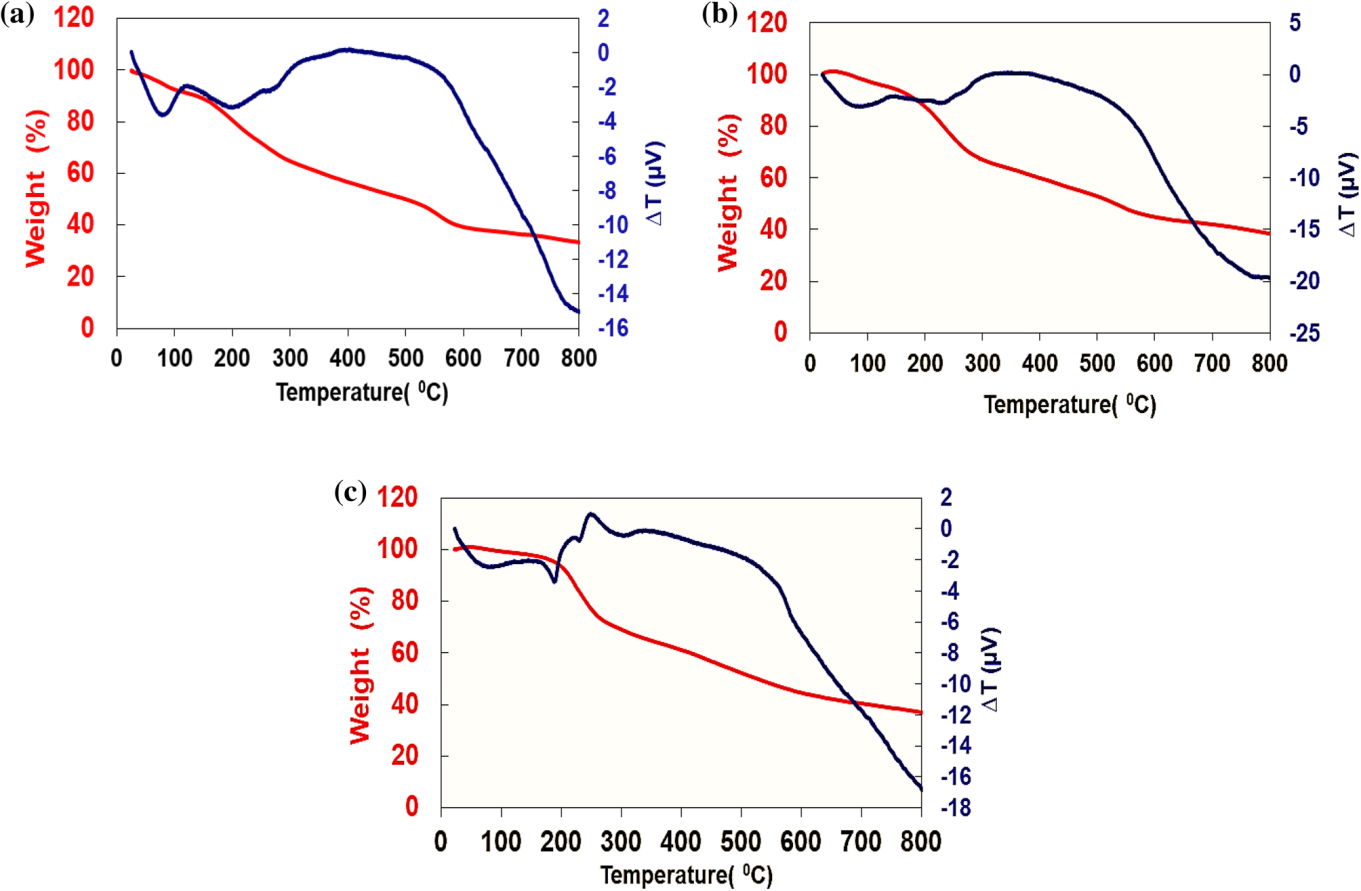
TGA and DTA profiles of (**a**) UiO-66-NH_2_, (**b**) UiO-66-NH_2_@cyanuric chloride, and (**c**) UiO-66-NH_2_@cyanuric chloride@5-amino tetrazole.

The TGA and DTA profile of UiO-66-NH_2_ is represented in Fig. 5a. The TGA curve exhibits a two-step weight loss. The first step occurs during the first 280 °C, concerned with the loss of adsorbed gas and coordinated hydroxyl groups to the zirconium cluster. The second step occurs between 280 and 800 °C, ascribed to the destruction of the frameworks. The final residue for the bare UiO-66-NH_2_ is 33.18%, which is the remaining zirconium oxide weight. The TGA curves of the UiO-66-NH_2_@cyanuric chloride and UiO-66-NH_2_@cyanuric chloride@5-amino tetrazole show the same weight loss profile as the UiO-66-NH_2_ with the final residue of 38.43% and 37.03% respectively, indicating the higher thermal resistance of modified MOF (Fig. 5b and c).

### Catalytic performance

After careful characterization of the as-synthesized UiO-66-NH_2_@Cyanuric Chloride@5-Aminotetrazole@Au_NPs_, we evaluate its catalytic performance for the A^3^-coupling preparation of a series of propargylamine products. To this end, we choose the reaction of benzaldehyde, piperidine, and phenylacetylene as the model reaction. We also examined the effects of the reaction time and temperature, the type of solvent, and the catalyst dosage on the progress of the reaction, and the results are depicted in Table 1. To find the best solvent, the progress of the A^3^-coupling reaction of the model reaction in the presence of the proposed catalyst in various solvents, including water, toluene, DMSO, DMF, CH_2_Cl_2_, MeCN, a mixture of EtOH and H_2_O, EtOH, tetrahydrofuran, and solvent-free condition was monitored. Despite the remarkable performance of our proposed method in solvent-free conditions, further studies were carried out with H_2_O (3 ml) as the solvent due to its green nature and ease of employment. Studies on the effect of the temperature on the progress of the reaction showed that the best results were obtained at 65 °C, and a further increase in the temperature did not increase the yield of the reaction. We monitored the progress of the reaction by the thin-layer chromatography technique, and we observed that at 65 °C in H_2_O, the reaction reached equilibrium after 150 min, 2.5 h. The ideal amount of the catalyst was obtained by tracking the progress of the reaction in the presence of various catalyst dosages. The results of this study indicated that 25 mg, 0.02 molar of the proposed catalyst is sufficient for the optimal progress of the A^3^-coupling reaction. So, based on these studies, 25 mg of UiO-66-NH_2_@Cyanuric Chloride@5-Aminotetrazole@Au_NPs_ catalyst at 65 °C in H_2_O and after 150 min results in the highest yields.

**Table 1:** The results of the optimization studies on the A^3^-coupling preparation of propargyl amines in the presence of UiO-66-NH_2_@Cyanuric Chloride@5-Aminotetrazole@Au_NPs_ catalyst.


Entry	Catal. (mg)	Solvent	T (°C)	Time (h)	Yield (%)
1	–	H_2_O	85	5	8
2	5	H_2_O	85	5	35
3	10	H_2_O	80	5	55
4	15	H_2_O	80	4	65
5	18	H_2_O	75	3.3	70
6	20	H_2_O	70	3.3	80
7	25	H_2_O	70	3.3	96
8	25	H_2_O	85	3.3	93
9	25	H_2_O	85	2.5	92
10	25	H_2_O	65	2.5	97
11	30	H_2_O	65	2.5	97
12	25	–	100	5	94
13	25	H_2_O	–	5	10
14	25	H_2_O	90	2.5	93
15	25	PhCH_3_	100	2.5	60
16	25	DMSO	100	2.5	62
17	25	DMF	100	2.5	65
18	25	CH_2_Cl_2_	45	2.5	30
19	25	MeCN	75	2.5	30
20	25	EtOH/H_2_O	–	2.5	90
21	25	EtOH	78	2.5	80
22	25	THF	60	2.5	35

Reaction conditions: 1 mmol of morpholine, 1 mmol of benzaldehyde, and 1.1 mmol of phenylacetylene.

After obtaining the optimum condition, we test the generality of our proposed method by synthesizing different propargyl amines from various precursors. In this context, diverse aldehydes and amines reacted with the phenylacetylene in the presence of the proposed catalyst and the optimum conditions. Table 2 summarizes the outcomes of this study. As this table indicates, the reaction proceeds in excellent yields in the presence of aromatic aldehydes containing electron donating or withdrawing groups in the aromatic ring's ortho or para positions. However, employing an aliphatic aldehyde resulted in lower yields. We used three different secondary amines in this reaction, and as shown in Table 2, the change in the amine did not affect the yield of the reaction. These results indicate the excellent performance of the UiO-66-NH_2_@Cyanuric Chloride@5-Aminotetrazole/Au_NPs_ as a catalyst for the promotion of the A^3^-coupling preparation of the propargyl amines.

**Table 2:** Preparation of various propargyl amines under optimum conditions.


Entry	Aldehyde	Amine	Product	Time (h)	Yield (%)
1				2.5	95
2				2.5	97
3				2.5	91
4				2.5	90
5				2.5	93
6				2.5	95
7				2.5	92
8				2.5	91
9				2.5	92
10				2.5	94
11				2.5	90
12				2.5	95
13				2.5	93
14				2.5	92
15				2.5	94
16				2.5	93
17				2.5	95
18				2.5	97
19				2.5	98
20				2.5	79

Reaction conditions: 1 mmol of morpholine, 1 mmol of benzaldehyde, and 1.1 mmol of phenylacetylene.

To see if the modulation of the microenvironment around the Au NPs by PSM of UiO-66-NH_2_ affects the promotion of the A^3^-coupling reaction, we monitored the formation of all the synthesized propargyl amine derivatives in the presence of Au NPs decorated UiO-66-NH_2_, UiO-66-NH_2_@Cyanuric Chloride, and UiO-66-NH_2_@Cyanuric Chloride@5-Aminotetrazole (Table 3). These tests were performed at the optimum conditions (25 mg, 0.02 mol catalyst, 65 °C, H_2_O, 150 min or 2.5 h) to compare the results. Table 3 indicates that each step of the PSM successfully modified the electronic structure of the chemical surrounding of the Au NPs, resulting in the improvement of the yield of the reaction. Based on this study, UiO-66-NH_2_@Cyanuric Chloride@5-Aminotetrazole/ Au_NPs_ is the optimum catalyst for promoting propargyl amines preparation via the A^3^-coupling reaction Table S1.

**Table 3:** Comparison of the catalytic performance of the proposed catalyst with some related reports in the *literature.*

Catalyst	Catal. (% mol)	Solvent	T (°C)	Time (h)	Yield %	References
Cu/Al/Oxide mesoporous	0.12	Toluene	90	22	94	^46^
Cu@N-rGo	–	–	70	8	76	^47^
Au-NPs	10	THF	75	5	92	^48^
Fe_3_O_4_/PT/Au	0.01	H_2_O	80	24	90	^49^
Au@SH-CNC	4.4	CHCl_3_	80	24	93	^50^
Au-NCs@Triazine-COP	0.8	CHCl_3_	60	6	87	^51^
Fe_3_O_4_@*R. tinctorum*/Ag NPs	0.1	H_2_O	80	8	96	^52^
AgI nanoparticles in/N_2_	1.5	H_2_O	100	20	96	^53^
Ag-NaY	5	–	100	15	81	^54^
ZnO-IL/Ag NPs	2	H_2_O	100	3	92	^55^
PS–NHC–Ag(I), N_2_	2	–	50	5	92	^56^
AgNPs@g-C_3_N_4_ MW heating	1	H_2_O: EtOH	80	0.33	96	^57^
AuBr_3_	0.25	H_2_O	70	12	> 99	^58^
CuO NPs	8	Toluene	90	5	87	^59^
Cu-MCM-41	4	–	90	1.5	93	^60^
Fe_3_O_4_@Caff ‐ Au	0.025	H_2_O	r.t	1	96	^61^
Fe_3_O_4_@C-NHCS-Au0	0.8	CHCl_3_	60	12	93	^62^
UiO-66-NH_2_@Cyanuric Chloride@5-Aminotetrazole@Au-NPs	25 mg, 0.02 mol	H_2_O	65	2.5	97	This work

**Figure 6:**
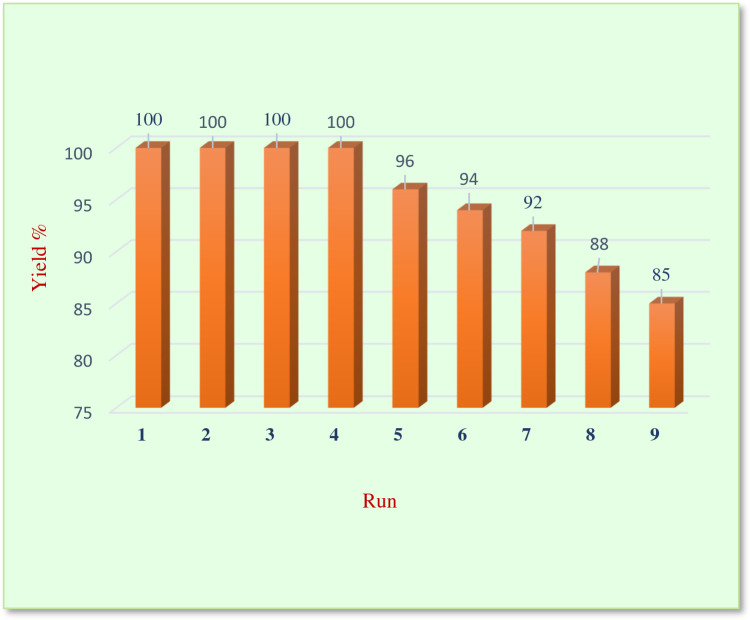
The recyclability of UiO-66-NH_2_@Cyanuric Chloride@5-Aminotetrazole@Au_NPs_ catalyst.

The catalyst's ability to be used several times for the same reaction is a defining factor for its industrial applications. To investigate the recyclability of our proposed catalyst, we separated the catalyst from the reaction medium by filtration and washed it with ethyl acetate several times for purification. Then, we applied the recycled catalyst to the model reaction for nine cycles and evaluated its performance. As shown in Fig. 6, no decrease in the catalyst performance was observed for four cycles, and even after six runs, its catalytic performance is still above 90% of its first use. This excellent reusability could be due to the high resistance nature of the UiO-66-NH_2_ MOF to the aquatic medium. Moreover, the post-synthesis modification of the UiO-66-NH_2_ filled the pores of MOF, limiting water molecules from accessing the zirconium cluster and increasing its stability.

Table 3 compares the catalytic performance of UiO-66-NH_2_@Cyanuric Chloride@5-Aminotetrazole@Au_NPs_ with some of the reported catalysts in the *literature* for the three-component preparation of propargyl amines. This table indicates that our proposed catalyst exhibits one of the highest reported yields. This could be due to the careful modification of the electronic structure of the UiO-66-NH_2_ via the post-synthesis modification process with nitrogen-rich ligands. The presence of cyanuric chloride and 5-Aminotetrazole alters the electronic structure of the UiO-66-NH_2_, leading to the boosting of the catalytic ability of the gold NPs in the final composite.

### Inductively coupled plasma-optical outflow spectroscopy examination (ICP-OES) comes out

The amount of gold (Au) loading measured via the result of ICP-OES to be used in the new catalyst was 0.84. Table 4 indicates the amount mol% of Au at every stage of reuses, which proves our proposed catalyst exhibits the highest activity reported yields. That was specify that the ICP investigation of the recuperated catalyst’s exercises to assess reused illustrated a really slight diminish in gold (Au) leaching.

**Table 4:** Mol% of Au per every step of reuses.

Entrance	Catalyst	Au (mol%)
1	Order 1	0.84
2	Order 2	0.81
3	Order 3	0.78
4	Order 4	0.71
5	Order 5	0.67
6	Order 6	0.6
7	Order 7	0.55
8	Order 8	0.5
9	Order 9	0.45

## Conclusion

In this report, the UiO-66-NH_2_ was chosen as a support for the heterogenization of the Au NPs due to its high potential for PSM, surface area, and inherent structural resistance. PSM of the MOF was carried out by a step-by-step strategy, in which a series of *N*-rich organic compounds were coordinated to the NH_2_ groups of the organic ligand of the MOF. The resulting catalyst was hired to promote the A^3^-coupling reaction, which showed superior performance. The results of this study indicate that such high efficiency is a result of the modulation of the microenvironment of the gold NPs. The proposed catalyst exhibited superior recycling performance due to the inherent resistance of the UiO-66-NH_2_ MOF and the induced resistance due to the PSM. Additionally, the proposed catalyst was reusable up to nine times.

## Supplementary Information

Supplementary Information.

## Data Availability

All data generated or analyzed during this study are included in this published article (and its Supplementary information files).
