# Supplementary material for: Gold nanoparticle decorated post-synthesis modified UiO-66-NH2 for A3-coupling preparation of propargyl amines
Source: Sci Rep. 2023 Jun 3;13:9051. doi: 10.1038/s41598-023-35848-4 (PMC10239494; doi:10.1038/s41598-023-35848-4)
Supplement: Supplementary file 1 — Supplementary Information. [file 41598_2023_35848_MOESM1_ESM.docx]

**Supporting information**

**Gold nanoparticle decorated post-synthesis modified** **UiO-66-NH_2_ for A^3^-coupling preparation of propargyl amines**

Leila Mohammadi, ^a^ Reza Taghavi, ^b^ Mojtaba Hosseinifard, ^c,^* Mohammad Reza Vaezi, ^a,^* Sadegh Rostamnia ^b^

*^a^Department of Nano Technology and Advanced Materials, Materials and Energy Research Center, Karaj, Iran.* *E-*mail: [*l.mohammadi3790@gmail.com*](mailto:l.mohammadi3790@gmail.com)*, :* [*m_r_vaezi@merc.ac.ir*](mailto:m_r_vaezi@merc.ac.ir)

*^b^ Organic and Nano Group (ONG), Department of Chemistry, Iran University of Science and Technology (IUST), PO BOX 16846-13114, Tehran, Iran. E-mail: reza.taghavee@gmail.com ,* [*rostamnia@iust.ac.ir*](mailto:rostamnia@iust.ac.ir)

*^c^ Department of Energy, Materials and Energy Research Center, Karaj, Iran. E-mail: m.hosseini@merc.ac.ir*

Table S1. investigation of the effect of PSM on catalyst efficiency

| Yield (%) for UiO-66-NH_2_@Cyanuric chloride@5-Aminotetrazole@Au  Final Catalyst | Yield (%) for UiO-66-NH_2_@Cyanuric Chloride@Au | Yield (%) for UiO-66-NH_2_@Au | Product | Entry |
| --- | --- | --- | --- | --- |
| 95 | **79** | **78** | **** | **1** |
| 97 | **80** | **79** | **** | **2** |
| 91 | **76** | **76** | **** | **3** |
| 90 | **77** | **75** | **** | **4** |
| 93 | **77** | **76** | **** | **5** |
| 95 | **83** | **83** | **** | **6** |
| 92 | **80** | **79** | **** | **7** |
| 91 | **79** | **78** | **** | **8** |
| 92 | **79** | **78** | **** | **9** |
| 94 | **81** | **80** | **** | **10** |
| 90 | **79** | **78** | **** | **11** |
| 95 | **85** | **84** | **** | **12** |
| 93 | **76** | **75** | **** | **13** |
| 92 | **77** | **76** | **** | **14** |
| 94 | **79** | **79** | **** | **15** |
| 93 | **79** | **79** | **** | **16** |
| 95 | **84** | **83** | **** | **17** |
| 97 | **85** | **84** | **** | **18** |
| 98 | **85** | **85** | **** | **19** |
| 79 | **68** | **67** | **** | **20** |

Reaction conditions: 1 mmol of morpholine, 1 mmol of benzaldehyde, and 1.1 mmol of phenylacetylene.

1-(1-(2-nitrophenyl)-3-phenylprop-2-ynyl)morpholine

1 H NMR (400 MHz, CDCl_3_): δ 2.43 (s, 2H), 2.62 (s, 2H), 3.63 (s, 4H), 5.65 (s, 1H), 7.26-7.56 (m, 7H), 7.71 (d, J = 7.5 Hz, 1H), 7.95 (d, J = 7.2 Hz, 1H). 13 C NMR (100 MHz, CDCl_3_): δ 49, 57.3, 66.3, 81.4, 90.1, 121.9, 123.8, 127.9, 128.1, 128.3, 129.6, 130.9, 131.3, 131.6, 149.4.

2-(1,3-diphenylprop-2-yn-1-yl) piperidine

^1^H NMR (CDCl_3_, 400 MHz): δ 7.70-7.64 (m, 2H), 7.58-7.53 (m, 2H), 7.43-7.34 (m, 6H), 4.91 (s, 1H), 2.65 (t, 4H), 1.73-1.59 (m, 4H), 1.50-1.48 (m, 2H). ^13^C NMR (CDCl_3_, 100 MHz): δ 138.00, 131.85, 128.74, 128.33, 128.19, 128.15, 127.68, 123.20, 88.08, 85.69, 62.33, 50.63, 25.94, 24.31.

3-(3-phenyl-1-(p-tolyl)prop-2-yn-1-yl)piperidine

^1^H NMR (CDCl_3_, 400 MHz): δ 7.59-7.54 (m, 4H), 7.39-7.35 (m, 3H), 7.22 (d, 2H, J=8), 4.89 (s, 1H), 2.66 (t, 4H), 2.40 (s, 3H), 1.73-1.60 (m, 4H), 1.51-1.49 (m, 2H). ^13^C NMR (CDCl_3_, 100 MHz): δ 137.40, 134.90, 131.85, 128.87, 128.72, 128.33, 128.16, 123.27, 87.90, 85.96, 62.08, 50.61, 25.92, 24.34, 21.19.

4-(1-(4-methoxyphenyl)-3-phenylprop-2-yn-1-yl)piperidine

^1^H NMR (CDCl_3_, 400 MHz): δ 7.59-7.55 (m, 4H), 7.38-7.36 (m, 3H), 6.94 (d, 2H, *J*=8), 4.80 (s, 1H), 3.86 (s, 3H), 2.60 (br, 4H), 1.67-1.47 (m, 6H). ^13^C NMR (CDCl_3_, 100 MHz): δ 159.00, 131.83, 130.67, 129.71, 128.31, 128.05, 123.41, 113.41, 87.65, 86.43, 61.80, 55.31, 50.64, 26.20, 24.51.

5-(1,3-diphenylprop-2-yn-1-yl) pyrrolidine

^1^H NMR (CDCl_3_, 400 MHz): δ 7.66 (t, 2H), 7.55-7.53 (m, 2H), 7.45-7.34 (m, 6H), 5.06 (s, 1H), 2.85-2.72 (m, 4H), 2.03-1.82 (m, 4H). ^13^C NMR (CDCl_3_, 100 MHz): δ 138.69, 131.84, 128.51, 128.37, 128.34, 128.03, 127.87, 123.03, 87.29, 86.07, 58.97, 50.19, 23.52.

6-(1-(4-bromophenyl)-3-phenylprop-2-yn-1-yl)piperidine

^1^H NMR (CDCl_3_, 400 MHz): δ 7.69 (d, 1H, *J*=8), 7.58-7.50 (m ,5H), 7.37 (t, 3H), 4.79 (s, 1H), 2.58 (t, 4H), 1.70-1.57 (m, 4H), 1.50-1.38 (m, 2H). ^13^C NMR (CDCl_3_, 100 MHz): δ 137.83, 131.85, 131.18, 130.24, 129.06, 128.36, 128.26, 88.30, 85.29, 61.79, 50.67, 26.14, 24.39.

7-(1-(naphthalen-3-yl)-3-phenylprop-2-ynyl) piperidine

^1^H NMR (400 MHz, CDCl_3_, ppm): δ 1.47-1.51 (m, 2H), 1.60-1.67 (m, 4H), 2.64 (t, 4H), 4.97 (s, 1H), 7.36-7.40 (m, 3H), 7.48-7.52 (m, 2H), 7.58-7.61 (m, 2H), 7.79 (dd, J^1^=J^2^=8.4 Hz, 1H), 7.85-7.91 (m, 3H), 8.11 (s, 1H); ^13^C NMR (100 MHz, CDCl_3_, ppm) d 24.4, 26.2, 50.8, 62.5, 86, 88.1, 123.3, 125.8, 125.9, 126.7, 127.2, 127.5, 127.7, 128.1, 128.12, 131.8, 132.9, 133.1, 136.3.

8-(3-phenyl-1-(o-tolyl) prop-2-yn-1-yl) morpholine

^1^H NMR (400 MHz, Chloroform-*d*) δ 7.76 – 7.71 (m, 1H), 7.58 – 7.53 (m, 2H), 7.39 – 7.32 (m, 3H), 7.23 (dd, *J* = 5.8, 3.7 Hz, 3H), 4.91 (s, 1H), 3.77 – 3.64 (m, 4H), 2.66 (t, *J* = 4.7 Hz, 4H), 2.51 (s, 3H). ^13^C NMR (75 MHz, Chloroform-d) δ 137.6, 135.8, 131.8, 130.8, 129.1, 128.4, 128.2, 127.9, 125.4, 123.1, 88.7, 85.1, 67.3, 59.9, 49.8, 19.2.

9-(3-phenyl-1-(p-tolyl) prop-2-yn-1-yl)morpholine

^1^H NMR (400 MHz, Chloroform-*d*) δ 7.50 (q, *J* = 3.5 Hz, 4H), 7.31 (t, *J* = 4.6 Hz, 3H), 7.20 – 7.13 (m, 2H), 4.74 (d, *J* = 3.4 Hz, 1H), 3.72 (d, *J* = 4.9 Hz, 4H), 2.62 (t, *J* = 4.6 Hz, 4H), 2.34 (s,3H).^13^C NMR (100 MHz, Chloroform-d) δ 137.5, 134.8, 131.8, 129.0, 128.6, 128.3, 128.2, 123.0, 88.3, 85.34, 67.2, 61.8, 49.9, 21.2.

10-(3-phenyl-1-(p-tolyl)prop-2-yn-1-yl)piperidine

^1^H NMR (CDCl_3_, 400 MHz): δ 7.59-7.54 (m, 4H), 7.39-7.35 (m, 3H), 7.22 (d, 2H, J=8), 4.89 (s, 1H), 2.66 (t, 4H), 2.40 (s, 3H), 1.73-1.60 (m, 4H), 1.51-1.49 (m, 2H). ^13^C NMR (CDCl_3_, 100 MHz): δ 137.40, 134.90, 131.85, 128.87, 128.72, 128.33, 128.16, 123.27, 87.90, 85.96, 62.08, 50.61, 25.92, 24.34, 21.19.

11-(1-(4-methoxyphenyl)-3-phenylprop-2-yn-1-yl) morpholine

^1^H NMR (300 MHz, Chloroform-*d*) δ 7.57 (t, *J* = 1.2 Hz, 1H), 7.54 (ddd, *J* = 6.0, 2.5, 1.6 Hz, 3H), 7.37 – 7.31 (m, 3H), 6.95 – 6.89 (m, 2H), 4.76 (s, 1H), 3.81 (s, 3H), 3.75 (td, *J* = 5.1, 3.0 Hz, 4H), 2.64 (dt, *J* = 5.2, 3.3 Hz, 4H). ^13^C NMR (75 MHz, Chloroform-*d*) δ 159.2, 131.8, 129.8, 129.7, 128.3, 128.2, 123.0, 113.6, 88.3, 85.40, 67.1, 61.4, 55.3, 49.8.

12-(3-phenyl-1-(pyrrolidin-1-yl) prop-2-ynyl) phenol

^1^H NMR (400 MHz, CDCl_3_): δ 1.87-1.89 (m, 4H), 2.78-2.88 (m, 4H), 5.29 (s, 1H), 6.82-6.87 (m, 2H), 7.19-7.27 (m, 1H), 7.34-7.37 (m, 3H), 7.51-7.54 (m, 3H). 13 C NMR (100 MHz, CDCl_3_): δ 23.8, 48.9, 57.1, 83, 89, 116.2, 118.9, 122.2, 122.6, 127.8, 128.4, 128.5, 128.8, 129.9, 131.9, 157.6.

13-(3-phenyl-1-(thiophen-2-yl)prop-2-yn-1-yl)morpholine

^1^H NMR (CDCl_3_, 400 MHz): δ 7.57-7.53 (m, 2H), 7.38-7.28 (m, 5H), 7.02-7.00 (m, 1H), 5.05 (s, 1H), 3.84-3.77 (m, 4H), 2.79-2.68 (m, 4H). ^13^C NMR (CDCl_3_, 100 MHz): δ 142.66, 131.89, 128.49, 128.38, 126.51, 126.38, 125.89, 122.66, 87.64, 84.25, 67.12, 57.79, 49.64.

14-(1-(2-chlorophenyl)-3-phenylprop-2-yn-1-yl) morpholine

^1^H NMR (CDCl_3_, 400 MHz): δ 7.81 (d, 1H, *J*=8), 7.56-7.53 (m, 2H), 7.48-7.42 (m, 1H), 7.39-7.28 (m, 5H), 5.18 (s, 1H), 3.79-3.75 (m, 4H), 2.73 (br, 4H). ^13^C NMR (CDCl_3_, 100 MHz): δ 135.51, 134.68, 131.86, 130.62, 129.95, 129.22, 128.44, 128.38, 126.42, 122.78, 88.42, 84.65, 67.11, 58.96, 49.85.

15-(3-phenyl-1-(thiophen-3-yl) prop-2-yn-1-yl)piperidine:

^1^H NMR (CDCl_3_, 400 MHz): δ 7.57-7.55 (m, 2H), 7.46 (s, 1H), 7.41-7.29 (m, 5H), 4.88 (s, 1H), 2.62 (br, 4H), 1.83-1.60 (m, 4H), 1.51-1.49 (m, 2H). ^13^C NMR (CDCl_3_, 100 MHz): δ 140.29, 131.86, 128.34, 128.15, 127.94, 125.39, 123.29, 123.26, 86.68, 86.22, 58.31, 50.62, 26.20, 24.48.

1-(1-(2-nitrophenyl)-3-phenylprop-2-yn-1-yl)morpholine

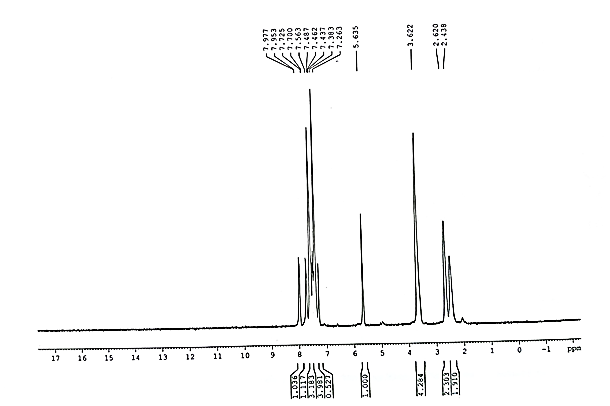


^1^HNMR

4-(1-(2-nitrophenyl)-3-phenylprop-2-yn-1-yl)morpholine


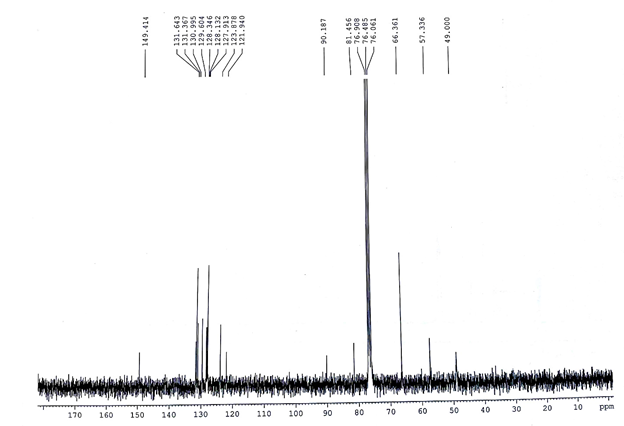


^13^CNMR

4-(1-(2-nitrophenyl)-3-phenylprop-2-yn-1-yl)morpholine

2 -(1,3-diphenylprop-2-yn-1-yl)piperidine

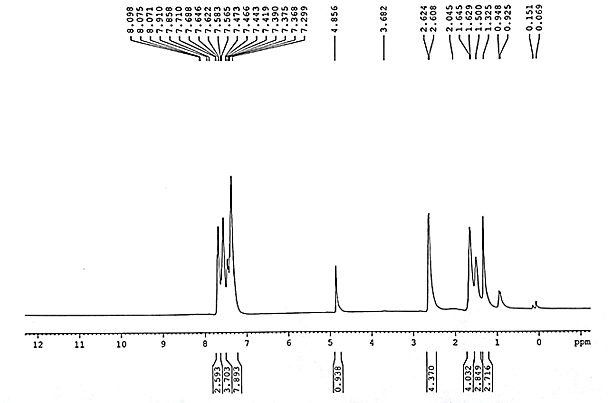


^1^HNMR

1-(1,3-diphenylprop-2-yn-1-yl)piperidine


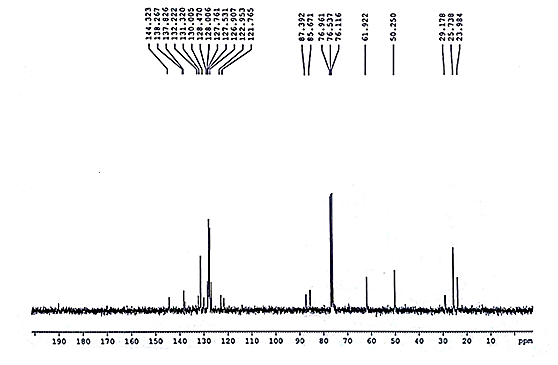


^13^CNMR

1-(1,3-diphenylprop-2-yn-1-yl)piperidine

3-(3-phenyl-1-(p-tolyl)prop-2-yn-1-yl)piperidine

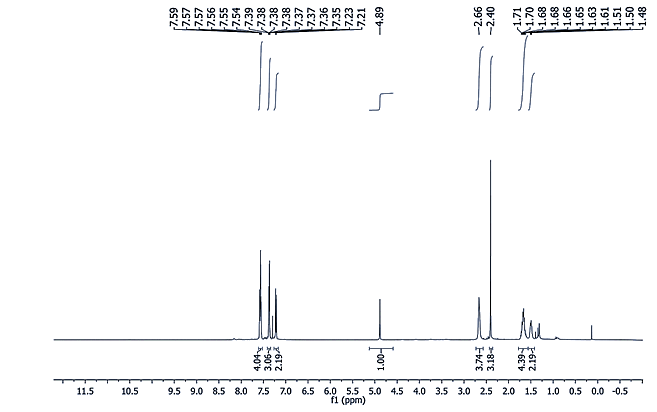


^1^H NMR

1-(3-phenyl-1-(p-tolyl)prop-2-yn-1-yl)piperidine


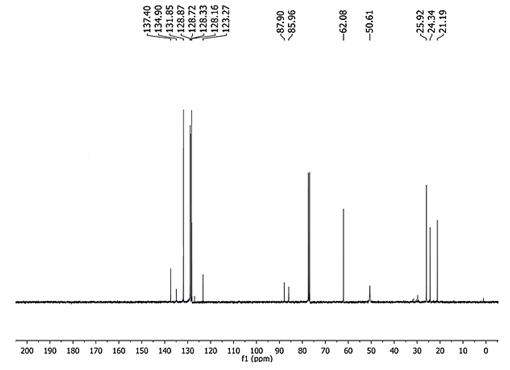


^13^C NMR

1-(3-phenyl-1-(p-tolyl)prop-2-yn-1-yl)piperidine

4-(1-(4-methoxyphenyl)-3-phenylprop-2-yn-1-yl)piperidine

^
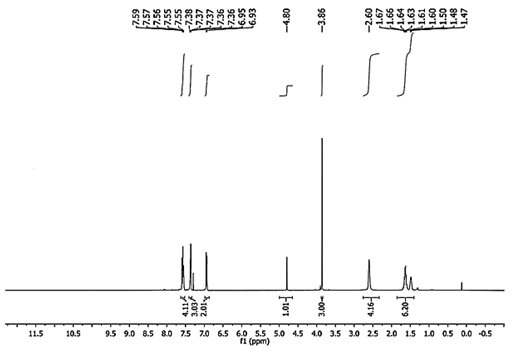
^

^1^H NMR

1-(1-(4-methoxyphenyl)-3-phenylprop-2-yn-1-yl)piperidine


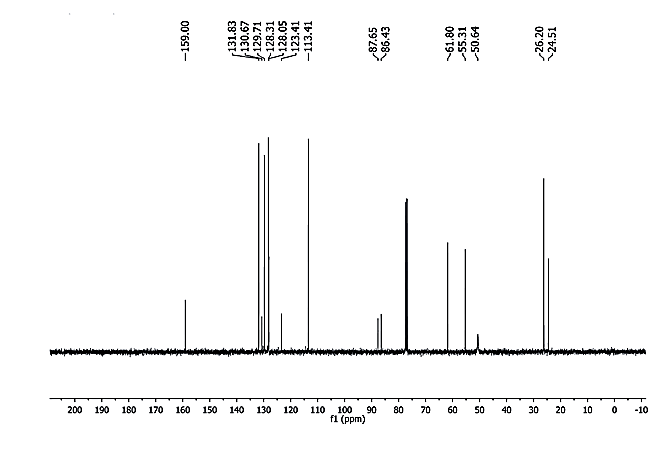


^13^CNMR

1-(1-(4-methoxyphenyl)-3-phenylprop-2-yn-1-yl)piperidine

5. 1-(1,3-diphenylprop-2-yn-1-yl)pyrrolidine

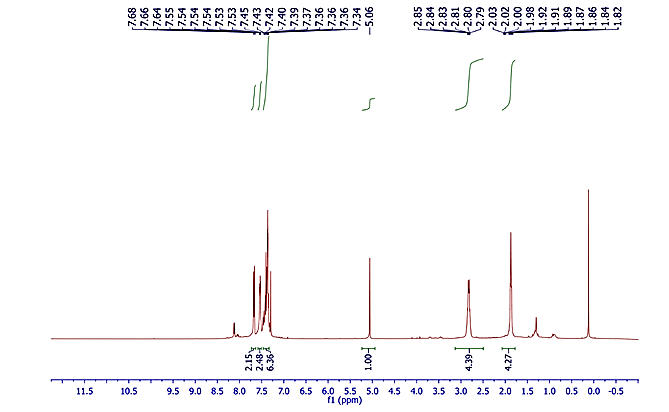


^1^H NMR

1-(1,3-diphenylprop-2-yn-1-yl)pyrrolidine


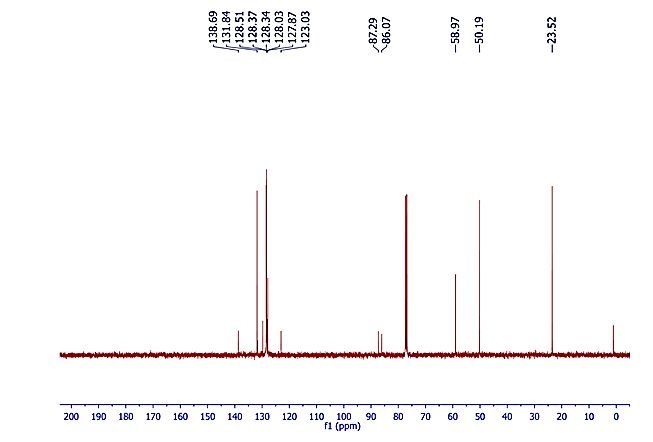


^13^C NMR

1-(1,3-diphenylprop-2-yn-1-yl)pyrrolidine


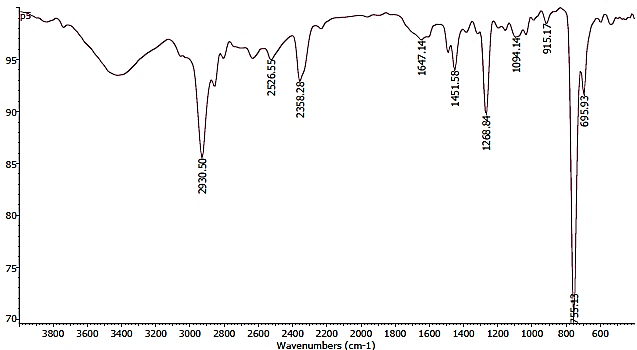


FT-IR

1-(1,3-diphenylprop-2-yn-1-yl)pyrrolidine

6-(1-(4-bromophenyl)-3-phenylprop-2-yn-1-yl)piperidine

^
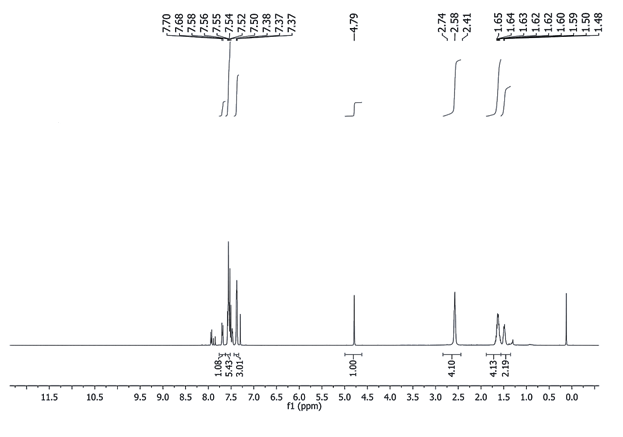
^

^1^H NMR

1-(1-(4-bromophenyl)-3-phenylprop-2-yn-1-yl)piperidine


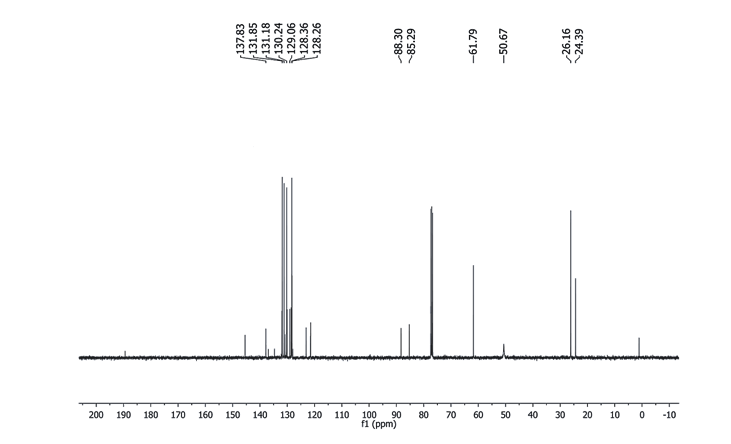


^13^C NMR

1-(1-(4-bromophenyl)-3-phenylprop-2-yn-1-yl)piperidine

7-(1-(naphthalen-1-yl)-3-phenylprop-2-yn-1-yl)piperidine

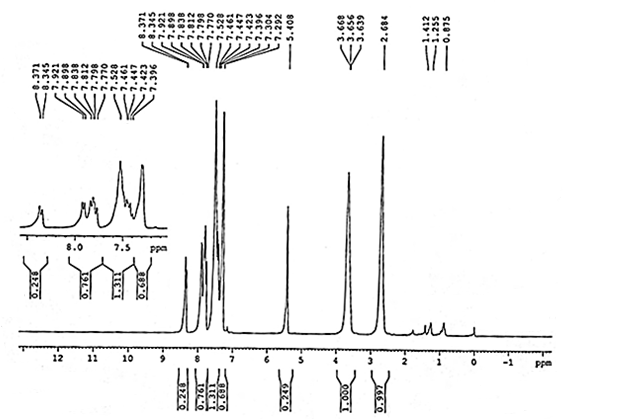


^1^H NMR

1-(1-(naphthalen-1-yl)-3-phenylprop-2-yn-1-yl)piperidine


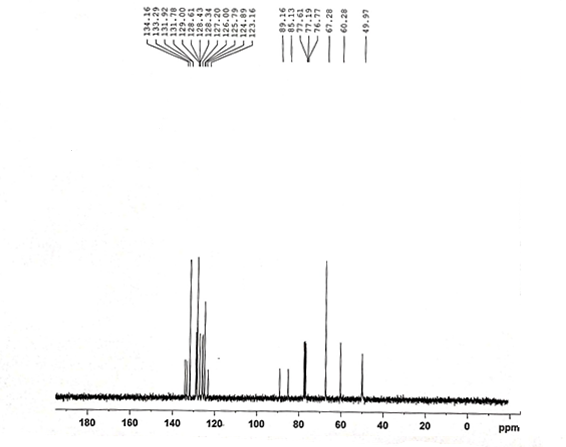


^13^C NMR

1-(1-(naphthalen-1-yl)-3-phenylprop-2-yn-1-yl)piperidine

8-(3-phenyl-1-(o-tolyl) prop-2-yn-1-yl)morpholine

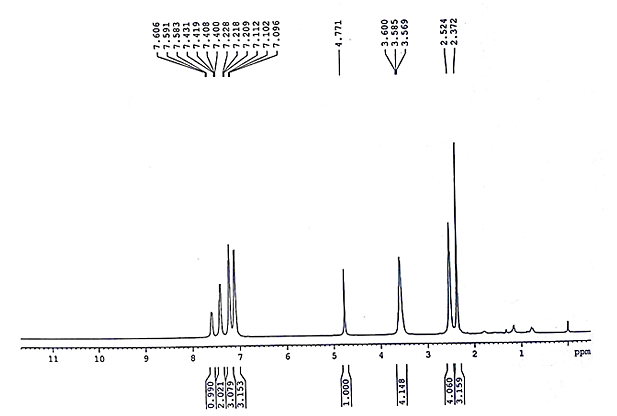


^1^H NMR

4-(3-phenyl-1-(o-tolyl) prop-2-yn-1-yl)morpholine


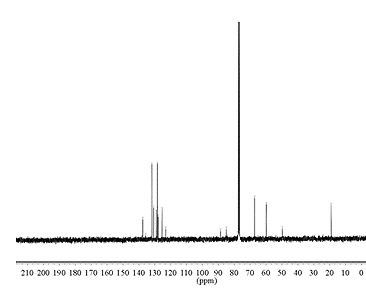


^13^C NMR

4-(3-phenyl-1-(o-tolyl) prop-2-yn-1-yl)morpholine

9-(3-phenyl-1-(p-tolyl)prop-2-yn-1-yl)morpholine

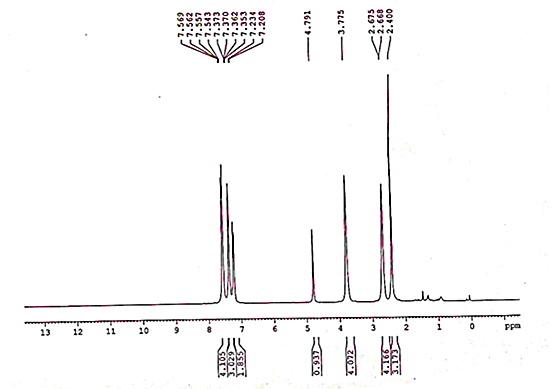


^1^H NMR

4-(3-phenyl-1-(p-tolyl)prop-2-yn-1-yl)morpholine


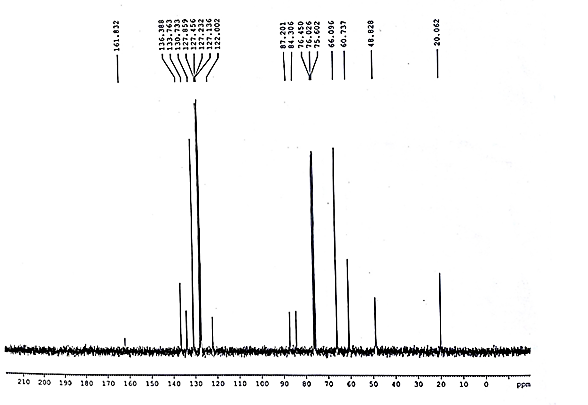


^13^C NMR

4-(3-phenyl-1-(p-tolyl)prop-2-yn-1-yl)morpholine

10-(3-phenyl-1-(o-tolyl)prop-2-yn-1-yl)piperidine

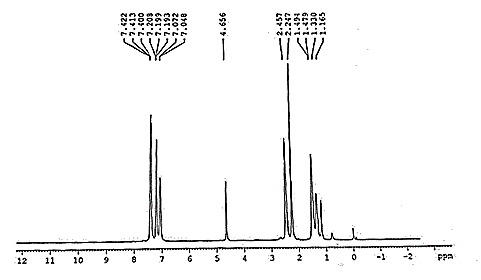


^1^H NMR

1-(3-phenyl-1-(o-tolyl)prop-2-yn-1-yl)piperidine


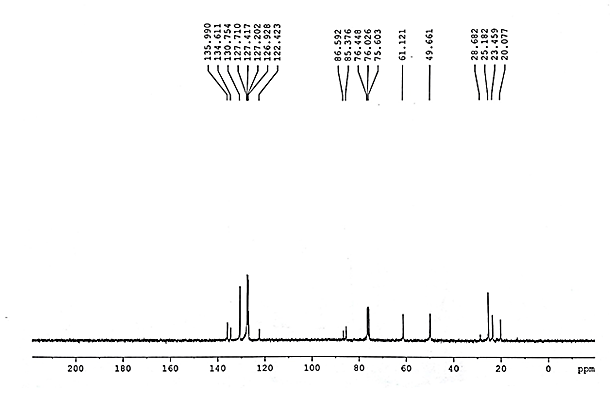


^13^C NMR

1-(3-phenyl-1-(o-tolyl)prop-2-yn-1-yl)piperidine

11-(1-(4-methoxyphenyl)-3-phenylprop-2-yn-1-yl)morpholine

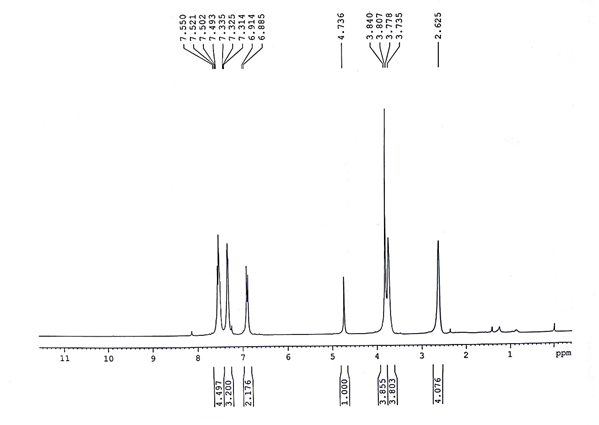


^1^H NMR

4-(1-(4-methoxyphenyl)-3-phenylprop-2-yn-1-yl)morpholine


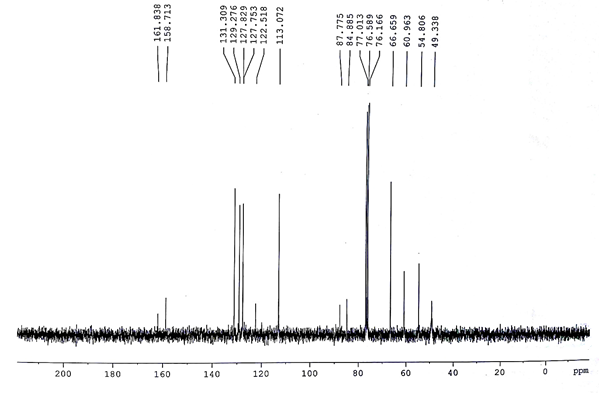


^13^C NMR

4-(1-(4-methoxyphenyl)-3-phenylprop-2-yn-1-yl)morpholine

12-(3-phenyl-1-(pyrrolidin-1-yl)prop-2-yn-1-yl)phenol

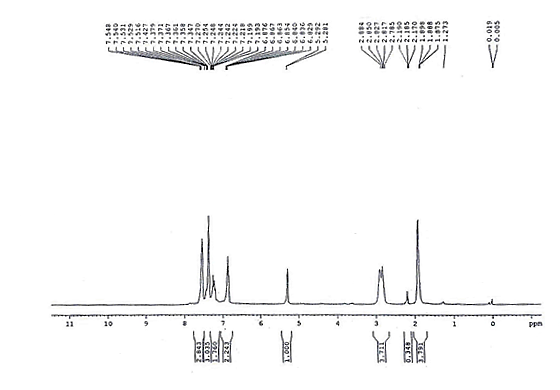


^1^H NMR

2-(3-phenyl-1-(pyrrolidin-1-yl)prop-2-yn-1-yl)phenol


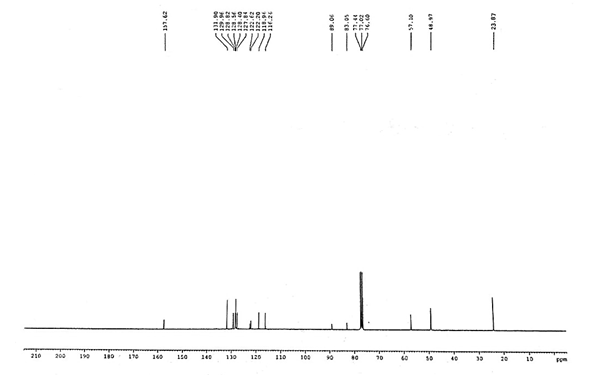


^13^C NMR

2-(3-phenyl-1-(pyrrolidin-1-yl)prop-2-yn-1-yl)phenol

13-(3-phenyl-1-(thiophen-2-yl)prop-2-yn-1-yl)morpholine

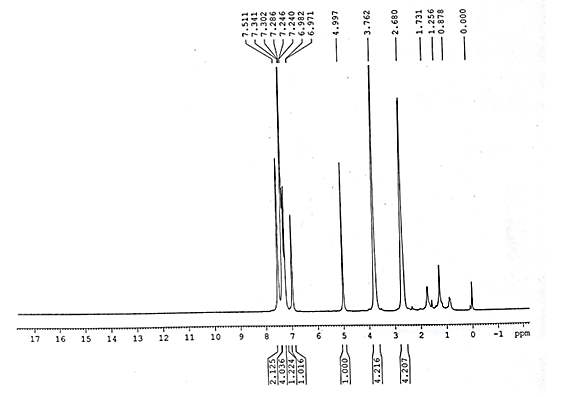


^1^H NMR

4-(3-phenyl-1-(thiophen-2-yl)prop-2-yn-1-yl)morpholine


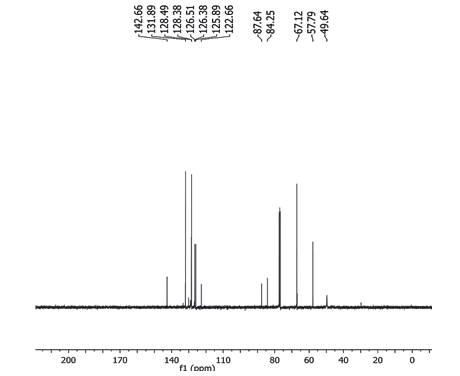


^13^C NMR

4-(3-phenyl-1-(thiophen-2-yl)prop-2-yn-1-yl)morpholine

14-(1-(2-chlorophenyl)-3-phenylprop-2-yn-1-yl)morpholine

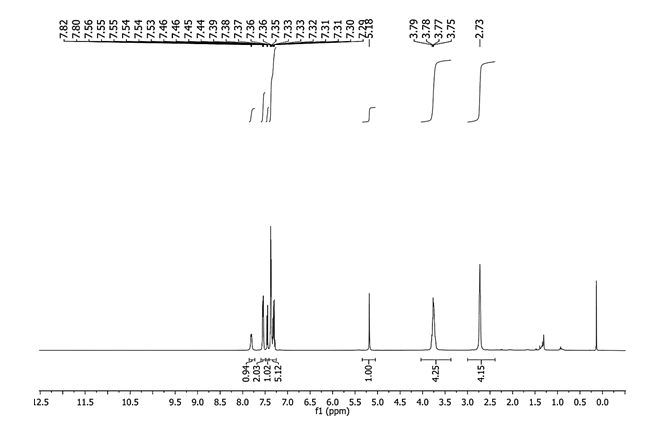


^1^H NMR

4-(1-(2-chlorophenyl)-3-phenylprop-2-yn-1-yl)morpholine


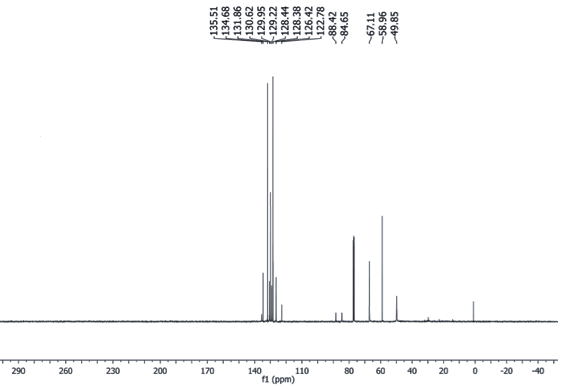


^1^C NMR

4-(1-(2-chlorophenyl)-3-phenylprop-2-yn-1-yl)morpholine

15-(3-phenyl-1-(thiophen-3-yl)prop-2-yn-1-yl)piperidine

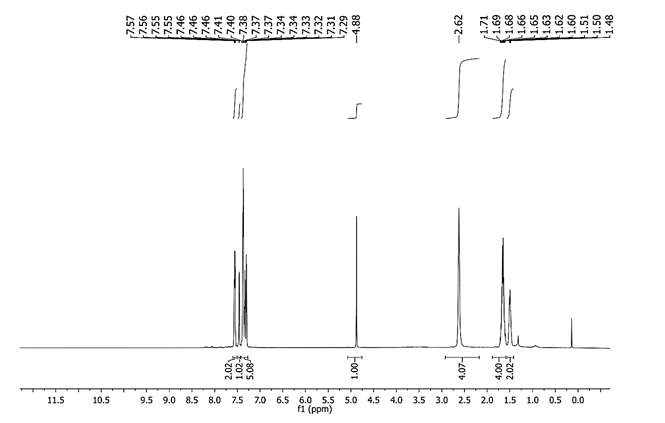


^1^H NMR

1-(3-phenyl-1-(thiophen-3-yl)prop-2-yn-1-yl)piperidine


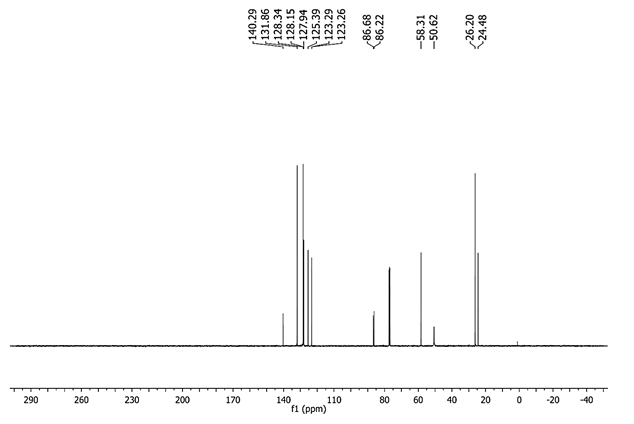


^13^C NMR

1-(3-phenyl-1-(thiophen-3-yl) prop-2-yn-1-yl) piperidine
